# Domestic Summary

**Published:** 1839-03-23

**Authors:** 


### DOMESTIC SUMMARY.

*Transylvania Medical School.*—We have received an extra of the Transylvania Journal of Medicine, containing the Annual Announcement of the Medical Department of the Transylvania University. A recent munificent endowment, by the council of the city of Lexington, will enable the trustees to erect a new hall and an infirmary. The library is to be immediately enlarged by purchases in the Eastern cities, and in Europe. The class of the past winter amounted to two hundred and eleven. The number of graduates at the commencement, March 9th, 1839, was fifty-one.

---

*Jefferson Medical College.*—At the annual commencement, held on the 5th inst., the degree of M. D. was conferred on eighty-eight gentlemen. The address was delivered by Prof. Calhoun.

---

*Massachusetts General Hospital Annual Report.*—As a statistical document it is important, and will doubtless be received, as those were which have preceded it, with satisfaction by the public. It is always gratifying to know how public charities are managed in the interior, which it is the object of these annual publications to explain. From January 1838, to January 1839, in the hospital in Boston, 380 patients were received; 174 were discharged well; 66 much relieved; 35 died; 3 eloped, and 48 were not relieved. The greatest number of patients in the house at any one time, 53. Expenses of the hospital for 1838, $13,096 54. Connected with this is the McLean Asylum for the Insane, at Charlestown, an admirably conducted institution. The whole number of patients remaining in the asylum at the commencement of the year, was 86; and there were received during the year 1838, 138 persons, which gives a total of 234 who enjoyed the benefits of this excellent charity. The year's expenditure, including $1,490 79 for a new building, was $29,096 71. The manner in which the trustees manage the two, is deserving of the highest commendation. Beside being liberally disposed, and always ready to co-operate with those who propose judicious improvements, they are not for ever interfering with the medical officers, which is the cause of so much ill temper, and operates so disastrously, in several of a kindred nature in neighbouring cities.—*Bost. Med. and Sur. Jour.*

